# Paradigm shift: Moving from symptom clusters to symptom networks

**DOI:** 10.1016/j.apjon.2021.12.001

**Published:** 2021-12-25

**Authors:** Zheng Zhu, Weijie Xing, Yan Hu, Bei Wu, Winnie K.W. So

**Affiliations:** Fudan University School of Nursing, Shanghai, China; Fudan University Centre for Evidence-based Nursing: A Joanna Briggs Institute Centre of Excellence, Shanghai, China; Fudan University School of Nursing, Shanghai, China; Fudan University Centre for Evidence-based Nursing: A Joanna Briggs Institute Centre of Excellence, Shanghai, China; Fudan University School of Nursing, Shanghai, China; Fudan University Centre for Evidence-based Nursing: A Joanna Briggs Institute Centre of Excellence, Shanghai, China; NYU Rory Meyers College of Nursing, New York City, NY, USA; The Nethersole School of Nursing, The Chinese University of Hong Kong, Hong Kong, China

Based on Dodd et al.'s definition, a “symptom cluster” refers to two or more concurrent symptoms that may or may not have the same etiology.^1^ Exploring symptom clusters is a classic analytical paradigm for dimension reduction to simplify complex scenarios in real-world clinical practice. However, Miaskowski et al., Zhu et al., and Barsevick's reviews identified that the combinations of symptoms may vary due to (1) the selection of symptoms included in the analysis; (2) the statistical methods used to decrease the dimensions of data; (3) stability over time; and (4) other covariates.^2^, ^3^, ^4^ In addition, there are an increased amount of opinions regarding whether the dimension reduction paradigm exploring symptom clusters suited to today's clinical practice with large amounts of big data? Shall we embrace the complexity of the connections among symptoms to solve real-world problems in symptom science?

In this comment, we indicate a primary direction for this research paradigm in symptom science using real-world data, which we call “symptom networks” or “symptomics”. Based on complex network theory, symptom networks can be understood as causal networks of mutually reinforcing symptoms by using network analysis. Symptom networks enable us to visualize and explore internal network structures of symptoms in a certain population, which may help researchers not only identify the association between symptom severities but also explore symptom mechanical indicators such as density, strength, closeness, and betweenness. Although the origin of the concept of symptom networks is in psychopathology, this paradigm has been used to capture complex relationships among symptoms of various chronic diseases in the last three years, including cancer and human immunodeficiency virus (HIV). Based on the types of data, contemporaneous networks, temporal networks, and dynamic networks could be conducted to describe either population-based or personalized network structures from a systematic perspective.

Symptom networks not only have the function of dimension reduction–like symptom clusters but can also guide health care providers and researchers in developing precise individualized interventions. The main features of the symptom network paradigm include the following: (1) Identifying the core symptom: Symptom networks enable researchers to find the most central symptom with the greatest importance in the network structure by assessing centrality measures such as strength, closeness, and betweenness. When analyzing symptoms from people with comorbidities, symptom networks may shed light on the mechanism by identifying the linkage of multiple groups or subgroups of symptoms via “bridge” symptoms without needing to allude to covariation between separate latent entities.

(2) Clustering symptoms: Symptom networks also have the same function of exploring symptom clusters as the traditional dimension reduction or clustering approach (e.g., principle components analysis, exploratory factor analysis, k-means). In addition, this new paradigm can not only detect symptom clusters for a specific disease but also be applied across multiple conditions at the same time based on large-scale symptom data. The changes in symptom clusters over time could also be explained and visualized by symptom networks.

(3) Symptom network density: Symptom network density is a more sensitive indicator than symptom severity and occurrence. Schweren et al. conducted a cohort study in 465 adolescents with depression and found that symptom network density could be regarded as a prognostic marker of treatment responses.^5^ Zhu et al. identified that in 2927 people living with HIV, a higher density indicated a higher probability of having comorbidities when symptom severity was at the moderate level and could not be differentiated by severity and occurrence.^6^

(4) Focusing on the microlevel interactions among symptoms: Analyzing symptom networks have become an available technique for zooming in on the microlevel interactions among symptoms. For a cohort design, understanding how the interactions change over time might have significant clinical implications. Rha and Lee analyzed the longitudinal data of 249 participants with cancer and found that the relationships among loss of appetite, taste change, nausea, and vomiting were closer after the start of chemotherapy, which suggested that the gastrointestinal cluster evolved during the chemotherapy cycle.^7^ For an experimental design, symptom networks also help researchers to understand complex causal mechanisms of interventions, which helps us to optimize the content of interventions to better target marker symptoms.

Overall, symptom clusters and symptom networks are two crucial paradigms in symptom science. Today, health care providers are facing real-world big data and patients with complex conditions. The symptom network paradigm additionally highlights the complexities of symptom interactions. This new paradigm may better suit today's real-world research and practice for symptom science. However, symptom networks are a relatively new paradigm. Additional studies are needed to explore the clinical significance of using symptom networks.

## Declaration of competing interest

None declared.
